# Helminths in Invasive Raccoons (*Procyon lotor*) from Southwest Germany

**DOI:** 10.3390/pathogens12070919

**Published:** 2023-07-08

**Authors:** Nico P. Reinhardt, Marion Wassermann, Jessica Härle, Thomas Romig, Lina Kurzrock, Janosch Arnold, Ernst Großmann, Ute Mackenstedt, Reinhard K. Straubinger

**Affiliations:** 1 Bacteriology and Mycology, Institute for Infectious Diseases and Zoonoses, Department of Veterinary Sciences, LMU Munich, 85764 Oberschleißheim, Germany; r.straubinger@lmu.de; 2 Parasitology Unit, Institute of Biology, University of Hohenheim, 70599 Stuttgart, Germany; marion.wassermann@uni-hohenheim.de (M.W.); jessica.haerle@yahoo.de (J.H.); thomas.romig@uni-hohenheim.de (T.R.); mackenstedt@uni-hohenheim.de (U.M.); 3 IDEXX Laboratories, Vet Med Labor GmbH, 70806 Kornwestheim, Germany; lina-kurzrock@idexx.com; 4 Wildlife Research Unit, Agricultural Centre Baden-Wuerttemberg (LAZBW), 88326 Aulendorf, Germany; janosch.arnold@lazbw.bwl.de; 5 Aulendorf State Veterinary Diagnostic Centre (STUA), 88326 Aulendorf, Germany; ernst.grossmann@stuaau.bwl.de

**Keywords:** nematodes, cestodes, trematodes, *Baylisascaris procyonis*, pathogen pollution, One Health

## Abstract

As hosts of numerous zoonotic pathogens, the role of raccoons needs to be considered in the One Health context. Raccoons progressively expand their range as invasive alien species in Europe. This study aimed to investigate the intestinal helminth fauna of raccoons in Baden-Wuerttemberg, Germany, as no such screening had ever been conducted there. In total, we obtained 102 animals from hunters in 2019 and 2020. Intestinal helminths were retrieved using the SSCT (segmented sedimentation and counting technique) and identified morphologically and by PCR-based Sanger sequencing. Fecal samples were assessed using the ELISA PetChek^TM^ IP assay (IDEXX, Germany) and flotation technique. The artificial digestion method was employed for analyzing muscle tissue. We detected species of four nematode genera (*Baylisascaris procyonis*, *Toxocara canis*, *Capillaria* spp., and *Trichuris* spp.), three cestode genera (*Atriotaenia* cf. *incisa/procyonis*, *Taenia martis*, and *Mesocestoides* spp.), and three trematode genera (*Isthmiophora hortensis/melis*, *Plagiorchis muris*, and *Brachylaima* spp.). *Echinococcus* spp. and *Trichinella* spp. were not found. The invasive behavior and synanthropic habits of raccoons may increase the infection risk with these helminths in wildlife, domestic and zoo animals, and humans by serving as a connecting link. Therefore, it is crucial to initiate additional studies assessing these risks.

## 1. Introduction

Ecosystems and life on earth, in general, are severely threatened by human-induced alterations that have led to a global climate crisis and the beginning of the sixth mass extinction event [1,2]. The convergence of both events leads to mutually adverse effects for One Health, partly attributable to the escalating environmental pollution and distribution of (non-)zoonotic pathogens [3,4,5,6]. The environment, wildlife, humans, and their domesticated animals are intricately interconnected through their diverse roles in maintaining and transmitting parasitic infectious diseases [2,5,7]. Considering the bidirectional transmission of parasites, with a predominant flow from wildlife to domestic animals, and the reverse direction, it is imperative to view at these pathogens and their respective hosts from a One Health perspective [5,7,8].

The raccoon, an extensively discussed mesocarnivore in this context, is recognized as a host of numerous pathogens and progressively expands its range as an invasive alien species (IAS) in Asia and Europe [9,10,11,12,13,14]. Since 2016, raccoons have been listed as IAS on the Union list of the Regulation (EU) No 1143/2014. In Germany, raccoons are found in different population densities across all 16 federal states, with two primary distribution areas in central and northeastern Germany. By the mid-21st century, raccoons are anticipated to populate most of the country [12,13]. The hunting bag of raccoons, especially in the northeastern area of Baden-Wuerttemberg (BW), has increased considerably. The number of hunted raccoons during the 2010/11 hunting season was 339, while in 2020/21, this figure had increased to 4015 [15]. According to Fischer et al. [12], the hunting bag data represents roughly 10% of the population. Based on this estimate, the total number of raccoons in BW is approximately 40,000. Genetic analyses have revealed that the raccoon population in BW primarily originates from Hessian raccoons, with some contribution from populations in the Harz and Rhineland-Palatinate regions [13,16,17].

Raccoons, being opportunistic generalists, inhabit diverse environments, frequently favoring locations close to water resources [18,19,20]. From an epidemiological standpoint, their synanthropic lifestyle holds particular significance as it contributes to reduced territorial ranges and increased population densities. This is primarily attributed to the benefits of abundant food resources and diverse shelter options [10,19,21,22]. Overall, raccoons can negatively impact native wildlife species by competing with and preying on them. Furthermore, raccoons pose a threat to the health of wildlife, domestic animals, and the public as reservoirs for various pathogens [14,21,23,24]. Raccoons defecate in specific places called latrines, which play a crucial epidemiological role, particularly in the life cycles of parasites, as many wild mammals and birds frequently visit and feed on undigested seeds found in raccoon feces [25,26]. Individual latrines are often used by various raccoons, which can increase the risk of intraspecies transmission [26]. The habitual proximity of latrines to humans and domestic animals poses an infection risk, particularly in urban areas [14,25,27]. Spatial overlaps of distinct species may enable pathogens to increase their abundance, broaden their host range and expand their geographic range [28].

Due to the synanthropic behavior of raccoons as an IAS, it is important to conduct investigations on infectious pathogens, particularly helminths. Raccoons have been extensively studied for their parasite infestations in their native range, with at least 100 different species of helminths from 65 distinct genera being documented globally [10,29]. A recently published study suggests that raccoons in Germany may also harbor a diverse range of metazoan parasites [10]. Furthermore, the occurrence and epidemiology of parasites can vary regionally due to different environmental factors [10,30]. However, only studies with limited oversight on parasite occurrence in raccoons in Germany, particularly in the southern regions, are available so far. Hence, we conducted a study to investigate the presence of intestinal helminths in raccoons of BW, considering that raccoons are significant hosts for various zoonotic helminths [7,14,16,31]. Such studies are necessary to better understand the raccoons’ role in pathogen transmission and its potential as an epidemiological link between infections in wild animals, domestic animals, and humans [3,6,7,9,14,19].

## 2. Materials and Methods

### 2.1. Sample Collection

**Figure 1:**
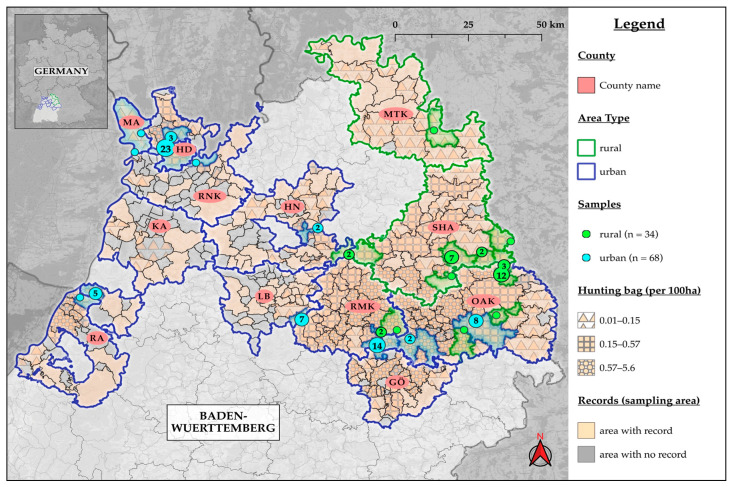
Map of raccoon sampling sites [9]. The illustration depicting the cumulative areas where raccoons were observed (records) based on survey records and hunting bag data of 2019 obtained from the Wildlife Research Unit at Agricultural Centre Baden-Wuerttemberg (LAZBW, Aulendorf, Germany). The hunting bag describes the number of raccoons hunted per 100 ha. MA (Mannheim), HD (Heidelberg), RNK (Rhein-Neckar-Kreis), RA (Rastatt), KA (Kreis Karlsruhe), HN (Kreis Heilbronn), LB (Kreis Ludwigsburg), RMK (Rems-Murr-Kreis), GÖ (Kreis Göppingen), MTK (Main-Tauber-Kreis), SHA (Kreis Schwäbisch Hall), OAK (Ostalbkreis).

We collected 102 free-ranging raccoons to analyze them for intestinal helminths. The collection of animals was between August 2019 and November 2020 during the hunting season for raccoons in BW (§10 DVO JWMG 2015, of 25 February 2018), except for one individual that had already been road-killed in May. The sampling area covered 27 municipalities in 12 counties in the northern part of BW. These counties included the City of Heidelberg (HD) and Mannheim (MA) as well as the districts Ostalbkreis (OAK; Ellenberg, Westhausen, Aalen, Lorch, Essingen, Schwäbisch Gmünd), Schwäbisch Hall (SHA; Fichtenau, Frankenhardt, Stimpfach, Bühlerzell, Kreßberg, Mainhardt), Rems-Murr-Kreis (RMK; Alfdorf, Plüderhausen), Karlsruhe (KA; Rheinstetten), Ludwigsburg (LB; Remseck am Neckar), Main-Tauber-Kreis (MTK; Weikersheim), Heilbronn (HN; Weinsberg, Wüstenrot), Rhein-Neckar-Kreis (RNK; Schriesheim, Dossenheim, Neckargemünd), Rastatt (RA; Durmersheim), Göppingen (GÖ; Börtlingen, Birenbach) (Figure 1). More than ten raccoons were collected in each of four counties (HD, OAK, SHA, RMK), five to nine from KA and LB, and less than five animals each from MTK, GÖ, HN, RNK, MA, and RA. The municipalities in which the hunting sites are located were classified as rural or urban areas based on the Federal Office for Building and Regional Planning (BBSR) classification [32,33]. The carcasses were stored at −20 °C until necropsy. No ethical approval was required for this study.

### 2.2. Necropsy

The necropsy of the carcasses was executed under BSL3 conditions at the Parasitology Unit of the University of Hohenheim. We evaluated the carcasses as fresh, minimally, or moderately autolytic. Heavily autolytic raccoons or incomplete animals were excluded. The animals’ weight, length, and sex were documented. Their age was determined as either adult (>1-year-old) or juvenile (<1-year-old) based on various factors such as tooth development and wear, weight and length, reproductive status, and hunting date [9].

We removed the small intestine and opened it longitudinally. Macroscopically visible helminths were collected and preselected in nematodes, cestodes, and trematodes. The smaller helminths were extracted by the segmented sedimentation and counting technique (SSCT) and detected by microscopy [34]. Nematodes and cestodes were stored in distilled water and then rapidly frozen at −80 °C. Trematodes were preserved in 80% ethanol. Fecal samples from the rectum were collected and stored at −80 °C for five days and subsequently transferred to −20 °C. The diaphragm pillar samples of the 101 dissected raccoons were examined for *Trichinella* spp. during necropsy by the compression method. Additionally, from a randomly selected 52 out of 101 raccoons, muscle samples of about 5 to 10 g of the diaphragm pillar and upper foreleg were collected and stored at −20 °C for further examination by the artificial digestion method at Aulendorf State Veterinary Diagnostic Centre (STUA, Germany, Aulendorf).

### 2.3. Parasitic Examinations

We identified nematode and cestode species by morphological molecular methods, whereas trematodes were assigned to a species exclusively based on morphology.

For morphological assessment and species identification, the trematodes were stained using Malzacher stain [35]. The prefixed trematodes were placed in alcoholic borax carmine, according to Grenacher, for 10 to 15 min, which stained the organs red. The stain was removed, and distilled water was applied to the trematodes for a differentiation step. As soon as the parenchyma was still slightly pink and the organs were red, the distilled water was removed, and astral blue was added. The predefined exposure time of ten minutes proved to be too long for small trematodes and was reduced to four minutes. The subsequent differentiation step also took longer and was adjusted in time to 15 to 20 min. Once the blue color had faded, the process continued. The stained objects were then dehydrated in an ascending ethanol series (50%, 70%, 90%, 95%, and 100%) for 10 min each. Trematodes were then placed in wintergreen oil, a methyl salicylic acid ester, for 30 min to make them more transparent. Finally, the trematodes were transferred on a slide into the inclusion medium, resin (Entellan^®^ new). After at least two days of hardening, the objects were measured and characterized according to Schell, S.C. [36] and Seo et al. [37] using photo-binoculars (ZEISS Axio Imager 2; Carl Zeiss Microscopy GmbH, Jena, Germany).

At STUA Aulendorf, 3 to 5 g of the collected feces per raccoon were tested for parasite eggs and larvae using the in-house flotation method followed by microscopical examination. A zinc chloride/saline solution (ZnCl_2_/NaCl) with a density of 1.35 g/mL was used for processing. Furthermore, 3–6 g per raccoon of the collected 52 muscle samples (diaphragm pillar, upper foreleg) were examined for *Trichinella* spp. at STUA Aulendorf using the artificial digestion methods according to the EU Regulation 2015/1375. To increase the test’s sensitivity, the samples were digested for 60 min and then sedimented for 40 min in the first step and 105 min in the second step. Then, 12 mL of sediment were diluted with 10 mL of water and examined for *Trichinella* spp. using the trichinoscope at 40–100 times magnification. In addition, 67 randomly selected fecal samples were tested for ascarids, hookworms, and whipworms using the ELISA-based PetChek^TM^ IP test kit (IDEXX, Kornwestheim, Germany) [38].

The intensity of infection, based on the number of parasite stadia found by visual examination during necropsy, was divided into three infestation groups: minor (≤5 cestodes, ≤5 nematodes, ≤5 trematodes), moderate (≤30 cestodes, ≤15 nematodes, ≤10 trematodes), and heavy infestation (>30 cestodes, >15 nematodes, >10 trematodes).

### 2.4. Nematode and Cestode Identification by PCR

#### 2.4.1. Sample Preparation

Small pieces of tissue (0.3–0.5 mm^3^) from each of the collected nematodes and cestodes were transferred to 200 μL PCR tubes containing 20–25 μL of 0.02 M NaOH and heated to 99 °C for 15 min [39]. The resulting lysate could be used directly as a template for the subsequent PCR assays.

#### 2.4.2. DNA Amplification and Sequencing

For species identification, the complete or partial mitochondrial (mt) NADH dehydrogenase subunit-1 (*nad1*) of the nematode and cestode samples were analyzed, and in the case of cestodes, an additional fragment of the mt cytochrome-c-oxidase subunit-1 (*cox1*). Nested PCRs were carried out with all samples; the primer combinations can be seen in Table 1.

The reaction volume for each sample was 25 µL for the first PCR and 50 µL for the nested PCR. The reaction mixtures consisted of 10 mM Tris–HCl (pH 8.3), 50 mM KCl, 2 mM MgCl_2_, 200 µM of each dNTP, 0.2 µM of each forward and reverse primer, and Taq polymerase (0.625 U for the first PCR, 1.25 U for nested PCR). As a template for the first PCR, 1 µL of the lysate was added to the reaction mixture, and for the nested, 1 µL of the primary PCR product was added. The PCRs were performed with an initial denaturation step at 95 °C (5 min), followed by 35 cycles of denaturation at 95 °C (30 s), annealing at 50–55 °C (30 s), elongation at 72 °C (1 min), and a final elongation step at 72 °C (5 min).

PCR results were detected on a 1.5% agarose gel stained with GelRed^TM^. PCR products were purified with the High Pure PCR Purification Kit (Roche, Mannheim, Germany) according to the manufacturer’s instructions and sent to Microsynth Seqlab GmbH (Göttingen, Germany) for sequencing. The electropherograms obtained by Sanger sequencing were analyzed using GENtle (Cologne, Germany) [40]. Sequences were compared with reference entries in the NCBI GenBank for species identification using the NCBI basic local alignment search tool (BLAST).

### 2.5. Statistics and Graphics

Microsoft^®^ Office Excel 16.63 was utilized for data management, graphics, and tables. QGIS 3.22 was used to create maps based on data on the occurrence of raccoons in each municipality provided by the Wildlife Research Center of the Agricultural Center Baden-Württemberg (WFS; LAZBW, Aulendorf, Germany). Statistical analyses were carried out using R (version 4.1.2) and RStudio (1 March 2023), but only for sample sizes ≥ 10 to ensure statistical validity. The prevalence of helminths in raccoons was calculated for the entire sample, as well as proportionally for age, sex, age–sex cohort, hunting year, and area type (rural vs. urban), along with the 95% Clopper–Pearson confidence intervals. Fisher’s exact test was used to determine any dependencies between the categories and a positive result for significant correlations. Differences in infestation levels within the category types (sex, age, area type) were calculated with the Kruskal–Wallis rank sum test. The Mann–Whitney U (MWU) test was used to compare body weight and length values between negative and positive results. A significance level of *p* ≤ 0.05 with a Bonferroni α adjustment was set for the statistical tests, and any significant effects are denoted with an asterisk (*).

## 3. Results

We found that 72.3% of the 101 raccoons (n = 73; 95% CI: 62.5–80.7) were positive for at least one helminth parasite in the examinations performed (Table 2). Cestodes represented the most common helminth group with 46.5% positive raccoons (n = 47; 95% CI: 36.5–56.7), ahead of nematodes with 31.7% (n = 32; 95% CI: 22.8–41.7) and trematodes with 15.8% positive individuals (n = 16; 95% CI: 9.3–24.4). Helminths detected in at least four raccoons are listed in Table 2. During the necropsy, 102 animals were examined, of which 80.4% (n = 82) were determined to be fresh, 12.7% (n = 13) were classified as minimally autolytic, and 5.9% (n = 6) were moderately autolytic. None of the collected animals was found to be heavily autolytic. The intestine of raccoon WB40 (male, adult, rural, Kreßberg, SHA) was incomplete due to hunting. Therefore, this raccoon was excluded from this study. This individual was examined exclusively by flotation; no parasites were found. When the sampled communities were classified as either urban or rural, it was found that 67.3% (n = 68) of the 101 raccoons were from urban areas, while 32.7% (n = 33) were from rural areas (Table 2 and Figure 1). Although detailed morphometric data were collected during the necropsy, they will not be further discussed since no significant correlation with parasite infection was identified.

### 3.1. Nematodes

**Figure 2:**
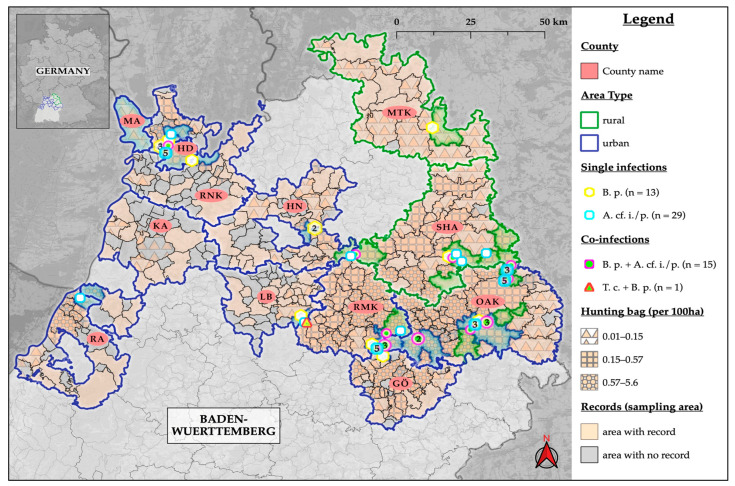
Map of helminth locations. The illustration depicting the cumulative areas where raccoons were observed (records) based on survey records and hunting bag data of 2019 obtained from the Wildlife Research Unit at Agricultural Centre Baden-Wuerttemberg (LAZBW, Aulendorf, Germany). The hunting bag describes the number of raccoons hunted per 100 ha. MA (Mannheim), HD (Heidelberg), RNK (Rhein-Neckar-Kreis), RA (Rastatt), KA (Kreis Karlsruhe), HN (Kreis Heilbronn), LB (Kreis Ludwigsburg), RMK (Rems-Murr-Kreis), GÖ (Kreis Göppingen), MTK (Main-Tauber-Kreis), SHA (Kreis Schwäbisch Hall), OAK (Ostalbkreis). *B. p.*, *Baylisascaris procyonis; A.* cf. *i./p.*, *Atriotaenia* cf. *incisa/procyonis; T. c., Toxocara canis*.

Nematodes were detected and identified in the intestines of 31.7% of 101 raccoons by the SSCT following PCR, the flotation technique, and PetChek^TM^ IP (IDEXX, Kornwestheim, Germany). Of these, 29 raccoons (28.7%; 95% CI: 20.1–38.6) were infected with *Baylisascaris (B.) procyonis*, one of them also with *Toxocara (T.) canis* (0.99%; 95% CI: 0.03–5.4) (Figure 2). Additionally, one raccoon with *Capillaria* spp. (0.99%; 95% CI: 0.03–5.4) and two with *Trichuris* spp. (1.98%; 95% CI: 0.2–7) were found.

By visual examination during necropsy and the SSCT, nematodes were identified in 29 raccoons. For each of the 29 infected raccoons, at least one of the examined nematodes could be assigned to a parasite species by sequencing. *Baylisascaris procyonis* was found in 28 animals and *T. canis* in one individual (WB64, male, adult, urban, Remseck am Neckar, LB). Ascarid eggs found in the *T. canis*-positive raccoon (WB64) were identified as *B. procyonis* by sequencing. Hence, raccoon WB64 had a dual infection with *T. canis* and *B. procyonis*. All *nad1* fragments showed 100% identity to the *B. procyonis* sequence JF951366 [41], except for the one nematode of WB64 (195 bp), which showed 99% similarity to a reference sequence from *T. canis* MN635719 [42].

Flotation detected ascarid eggs in ten raccoons that were also positive for other *B. procyonis* stadia. A single infection with *Capillaria* spp. was recorded exclusively in the flotation for WB67 (male, adult, urban, HD). Moreover, 24 of the 67 raccoons tested for ascarids with PetChek^TM^IP ELISA (IDEXX, Kornwestheim, Germany) were also positive for *B. procyonis* in the PCR assays. Only 8 of the 24 *B. procyonis*-positive raccoons were thus confirmed in the ELISA. In addition, *Trichuris* spp. were detected in two raccoons exclusively by PetChek^TM^IP ELISA (IDEXX, Kornwestheim, Germany). One of these (WB19, rural) was a juvenile female raccoon from Frankenhardt, SHA, and the other was a juvenile male individual from Lorch, OAK (WB96, urban).

No *Trichinella* spp. were found in the examinations using the artificial digestion or compression method. No significant dependencies of the tested categories with Fisher’s exact test between the positive and negative nematode groups could be found.

### 3.2. Cestodes

A total of 46.5% cestode-positive raccoons were exclusively detected by visual examination during necropsy and the SSCT (Table 2). The cestodes were determined based on morphological characteristics, and to verify these we used the described PCR tests with subsequent sequencing. Of the 60 cestode specimens analyzed via sequencing, 38 yielded sequences that could be evaluated.

We detected *Atriotaenia (A.)* cf. *incisa*/*procyonis* in 43.6% (n = 44; 95% CI: 33.7–53.8) of the 101 examined raccoons (Table 2 and Figure 2). The morphological examination allowed the identification of the cestodes as *Atriotaenia* spp. This was performed using written descriptions of the morphology of *Atriotaenia* spp., such as the characteristics of the scoleces [43]. No cestode other than *A. incisa* or *A. procyonis*, which resembles this genus, is known to infect raccoons. Of the 38 yielded gene sequences, 36 were identical to each other, including those of the morphologically determined cestodes. Consequently, all 36 identical sequences were assigned to either *A. incisa* or *A. procyonis*. The comparison of the most frequently encountered sequences with the references in the NCBI GenBank did not allow species identification. The approximately 730 bp-long *cox1* gene fragment showed the highest similarity with 82.8% to a *Dipylidium caninum* sequence (NC021145). Therefore, the complete *nad1* gene was also analyzed from some of these samples. This 900 bp-long sequence had the highest match of 87.9% with *Taenia crassiceps* (NC002547). Since genetic determination was not successful and it is almost impossible to distinguish morphologically between *A. procyonis* and *A. incisa*, the name *A.* cf. *incisa/procyonis* is used here [43]. The complete *nad1* and partial *cox1* gene *A.* cf. *incisa*/*procyonis* gene sequences were submitted to the NCBI GenBank under the Accession Numbers OR001745 for *nad1* and OR039311 for *cox1.* Cestodes in the total and *A.* cf. *incisa/procyonis*-positive raccoons originated more frequently from rural than urban areas with a significance of *p* = 0.02. No other significant dependencies of the tested categories with Fisher’s exact test between the positive and negative cestode groups could be found.

*Taenia (T.) martis* was found in 1.98% (n = 2; 95% CI: 0.2–7) of the 101 raccoons, of which WB37 (juvenile, male, urban, Aalen, OAK) was also infected with *A.* cf. *incisa*/*procyonis*. The other *T. martis*-positive raccoon was a male adult individual from a rural area (Bühlerzell, SHA; WB30). Species identification was performed by molecular means. Small fragments of the *cox1* (118 bp) and *nad1* (51 bp) genes were obtained. They were 100% congruent with the reference sequence of *T. martis* AB731758 [39]. Although the fragments were short, both could thus be clearly assigned to this species. Furthermore, we found *Mesocestoides* spp. in one (0.99%; 95% CI: 0.03–5.4) of the *A.* cf. *incisa*/*procyonis*-positive raccoons (WB93, female, adult, rural, SHA, Frankenhardt). Four scoleces were isolated from this raccoon and could be identified as *Mesocestoides* spp. based on their size and characteristic morphology. Neither the nested PCR with the *cox1* nor the *nad1* primers yielded evaluable results. Cestodes from two male adult raccoons (WB18 (rural, Frankenhardt, SHA) and WB94 (urban, Lorch, OAK)) could not be attributed morphologically or genetically to a specific species. No *Echinococcus* spp. were detected by the SSCT.

### 3.3. Trematodes

Trematodes were detected in 15.8% of the raccoons (n = 16; 95% CI: 9.3–24.4) by the SSCT (n = 14) and flotation (n = 2). Adult trematodes of half of the 14 positive raccoons were morphologically assigned to a genus or specific species, while this was not possible for the other 7 due to the poor condition of the worms. Two trematode infections were exclusively detected through egg flotation (WB52 and WB75), and the causative species, therefore, were not determined. The trematodes of 4% of the examined raccoons (n = 4; 95% CI: 1.1–9.8) were assigned to *Isthmiophora (I.) hortensis/melis*. Without genetic differentiation, *I. melis* and *I. hortensis* cannot be distinguished from each other. Two raccoons were male and adult (WB08, WB100) and two were male juvenile individuals (WB09, WB102; Table 2). Raccoons WB08 and WB09 originated from KA (Rheinstetten) and WB100 (Ellenberg), and WB102 (Lorch) from OAK. The latter two raccoons had a heavy infestation of *I. hortensis/melis,* with 30 individuals each. Raccoon WB10 (male, adult, urban) from Rheinstetten, KA was infected with *Plagiorchis muris* (0.99%; 95% CI: 0.03–5.4). In addition, *Brachylaima* spp. were detected in 1.98% of raccoons (n = 2; 95% CI: 0.2–7). One individual originated from Aalen, OAK (male, juvenile, urban, WB36) and the other animal from Weinsberg, HN (female, adult, urban, WB74). No significant dependencies of the tested categories with Fisher’s exact test between the positive and negative trematode groups could be found.

### 3.4. Intensity of Infestation and Co-Infections

**Figure 3:**
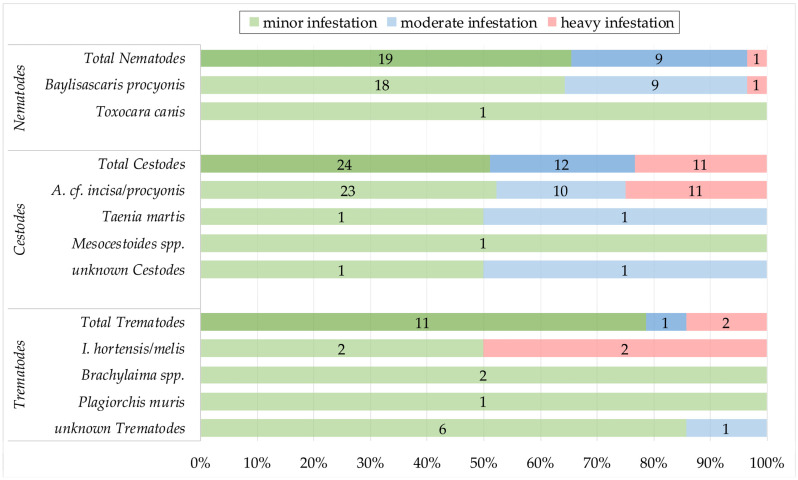
Infestation intensity of helminths based on morphological examination. The numbers represent raccoon counts per infestation level. *A.* cf. *incisa/procyonis*, *Atriotaenia* cf. *incisa/procyonis*; *I. hortensis/melis*, *Isthmiophora hortensis/melis*.

Based on visual examination during necropsy and the SSCT, the infestation levels of the individual helminths could be determined (Figure 3). Thus, of the 29 raccoons infested with nematodes, 65.5% had minor (n = 19; 95% CI: 45.7–82.1), 31% had moderate (n = 9; 95% CI: 15.3–50.8), and 3.4% had a heavy infestation (n = 1; 95% CI: 0.1–17.8). Of the 47 cestode-positive raccoons, 51.1% were minorly (n = 24; 95% CI: 36.1–65.9), 25.5% were moderately (n = 12; 95% CI: 13.9–40.3), and 23.4% were heavily infested (n = 11; 95% CI: 12.3–38.0). Of the 14 raccoons with trematodes, 78.6% had a minor infestation (n = 13; 95% CI: 49.2–95.3), 7.1% were moderately (n = 1; 95% CI: 0.2–33.9), and 14.3% were heavily infested (n = 2; 95% CI: 1.8–42.8) (Figure 3). No significant differences in infestation levels within the categories (sex, age, area type) were seen with the Kruskal–Wallis rank sum test.

Co-infections were detected in 32.9% of the 73 positive raccoons (n = 24; 95% CI: 22.3–44.9) (Table 3). The largest proportion, 21.9% of the positive raccoons (n = 16; 95% CI: 13.1–33.1), had a dual infection with nematodes and cestodes (Table 3). In two cases each, we found dual infections with nematodes and cestodes and with cestodes and trematodes. One raccoon, WB36 (female, juvenile, urban, Aalen, OAK), had a triple infection with all three parasite groups (Table 3).

## 4. Discussion

This study aimed to systematically investigate the occurrence of helminths of raccoons in BW, as no such screening had been carried out in the southwestern region of Germany. We found that 72.3% of 101 free-ranging raccoons were positive for at least one helminth parasite in the examinations performed (Table 2). Among the helminths detected, there were positive results for species of four nematode genera (*B. procyonis*, *T. canis*, *Capillaria* spp., and *Trichuris* spp.), three cestode genera (*A.* cf. *incisa/procyonis*, *T. martis* and *Mesocestoides* spp.), and three trematode genera (*I. hortensis/melis*, *Plagiorchis muris*, and *Brachylaima* spp.). The detected parasite diversity and presence of six potentially zoonotic helminths are relevant considering the One Health aspect. According to our findings, the raccoons’ propensity for spreading and synanthropic habits may lead to environmental pollution in BW with pathogens, including helminths [9]. Consequently, raccoons may serve as a transmission link between various animal groups, including wildlife, domestic and zoo animals, and humans, thereby elevating the risk of infection [3,6,12,14].

### 4.1. Methodological Limitations

The acquisition of raccoons was exclusively conducted in the northern half of BW, where the population density and hunting activity are highest (Figure 1). Therefore, our results are not representative of the entire region of southwestern Germany. The spatial and temporal distribution of raccoon carcasses and their submission may be biased due to complex biological factors (e.g., raccoon population dynamics) and human-related factors (e.g., hunters’ commitment in respective areas) [44]. To provide an overview of helminths in the raccoon population of BW, we restricted our sample size to approximately 100 animals. Based on a hunting bag of 4015 raccoons during the 2019/2020 hunting season, the estimated raccoon population in BW is estimated to be 40,000 animals [13,15]. With a sample size of 101 raccoons, only 3.3% of the hunting bag and 0.3% of the estimated population in BW were represented [9]. Higher numbers are required for accurate prevalence and population estimates. Two-thirds of the sampled raccoons were from urban areas, which is not surprising given the high raccoon densities in such areas and the densely populated sample area (Figure 1) [9]. The categorization of raccoons into urban and rural areas is a simplified classification, as it only considers the hunting community and not the individual raccoon’s spatial usage [9]. Home range sizes can vary based on factors such as season, habitat and area type (urban > rural), sex (male > female), and age (adult > juvenile) [21,23].

Helminth prevalence may be underestimated due to the natural decomposition processes of intestinal parasites. The freezing and thawing of the carcasses and samples may have damaged or even destroyed less persistent parasites such as flukes, small tapeworms [45], and some *Trichinella* spp. [46], leading to an underestimation of the overall infestation rate. For some cestodes and trematodes, the genera were determined exclusively by morphological and parasitological examinations. In this context, it should be mentioned that these are not completely reliable, and a genetic identification is preferred. To detect intestinal nematodes in raccoons, we used a combination of three methodological approaches (visual examination/SSCT, flotation, and ELISA-based PetChek^TM^ IP) to obtain the most accurate picture and prevalence of the nematode fauna [8,47,48], of which the ELISA test was only performed in 67 of the 101 raccoons. The intestinal collection of nematodes is the gold standard to obtain reliable prevalence values, including prepatent infections [8,48]. Prepatent infections, however, are not detected in fecal egg flotations, which can lead to an underestimation of the true prevalence [8,27,48]. In addition, male-only infections, insufficient fecal material, irregular shedding, and fluctuating amounts of eggs, as is the case in many intestinal helminths, e.g., *Baylisascaris* spp., can lead to false-negative results in the flotation [8,27]. Of the *B. procyonis*-positive raccoons by visual examination and the SSCT, only 35.7% (10/28) were also positive by egg flotation; thus, 64.3% of the positive samples remained undetected. The species of ascarid eggs in the flotation was not corroborated by PCR in the samples with *B. procyonis* individuals detected by visual examination and the SSCT.

The ELISA-based PetChek^TM^ IP assay detects antigens of (pre-)patent stadia of ascarids, hookworms, and whipworms in feces; however, it has not yet been validated for detecting *Baylisascaris* spp. [8,38]. The eight raccoons positive for ascarids detected by this ELISA were also positive for *B. procyonis* in the PCR assays. Thus, it is reasonable to assume that the ELISA is able to identify *B. procyonis*. However, 16 samples with *B. procyonis* could not be confirmed by this ELISA.

Hence, it is evident that the selection of methods can significantly impact the prevalence, which must be considered when evaluating them.

### 4.2. Nematodes

#### 4.2.1. *Baylisascaris procyonis*

*Baylisascaris procyonis* was found in 28.7% of 101 examined raccoons, making it the second most prevalent helminth species in this study. Previously reported prevalence values for *B. procyonis* in Germany vary significantly, ranging from 0% to 95% depending on the geographical region [10,16,25,49,50]. Raccoon populations in central Germany, including urban areas, especially exhibit remarkably high prevalence rates [10,25,49]. Prevalence rates in neighboring countries show considerable differences but tend to be lower than those in Germany [18,47,51,52]. The prevalence variations of *B. procyonis* in endemic areas are influenced by the migrated raccoons from founder populations, with additional ecological or geographical factors potentially playing a role [16,50]. The population growth and spatial expansion led to the mixing of founder populations and consequently to the spread of *B. procyonis* [17,30]. It is described that in BW, raccoons from the Hesse, Harz, and Rhineland-Palatinate regions had mixed, with a high prevalence of *B. procyonis* reported in Hessian and Harz raccoons [13,16,17,53]. Given that the raccoon population is in the early stages of spreading in BW, it can be assumed that *B. procyonis* prevalence rates will continue to increase. A total of 64.3% of the 28 raccoons that tested positive in the visual examinations and SSCT showed a mild infestation, 32.1% had a moderate infestation, and a heavy infection was observed in only 3.7% of the raccoons (Figure 3). These results contradict previous studies from Germany, which often reported heavy or moderate infestations [10,25]. However, it should be noted that the categorization criteria for infestation severity may differ between studies. Although many publications identified influencing factors, such as age, sex, land use, area type or season [8,20,22,54,55,56], this could not be detected in the present study.

The genus *Baylisascaris* comprises ten species, with *B. procyonis* being considered the most pathogenic in intermediate and accidental hosts [22,25]. *Baylisascaris procyonis* is an emerging zoonotic pathogen with an indirect life cycle involving raccoons as the final hosts, typically resulting in subclinical infections [2,57,58,59]. In addition, there are more than 130 intermediate or accidental hosts (rodents, birds, domestic animals, wild carnivores, primates, and humans) of this parasite, which can occasionally develop severe clinical signs resulting from visceral, ocular, or neural *larva migrans* syndrome [27,57,58]. Raccoons infected with *B. procyonis* can excrete over 200,000 EPG (eggs per gram of feces), which become infectious after 14 days and can remain infective in the environment for years under suitable moisture conditions [25,55,57,58]. Raccoons defecating on communal latrines, which are visited by various potential intermediate hosts and are an essential and persistent source of *B. procyonis* infections, lead to environmental contamination with this parasite [25,26,27]. Furthermore, it should be noted that domestic dogs not only serve as intermediate hosts for *B. procyonis* but can also act as alternative final hosts [4,8,27,58]. Therefore, in areas with high *B. procyonis* prevalence, dogs may pose a potential public health risk and may extend the range of environmental contamination [5,8,58]. Additionally, it should be noted that *B. procyonis* can negatively impact the populations of potentially endangered intermediate hosts, contributing to biodiversity loss [5,8,25,27,53,58]. In many zoos, *B. procyonis* infections are of increasing concern for various animal species [52,60,61]. The exposure to *B. procyonis* in zoological facilities appears to be real in BW, as indicated by the detection of *B. procyonis* eggs in the latrines of one out of six raccoon enclosures tested using the flotation method (Reinhardt, N. P.; unpublished). Zoological institutions in raccoon distribution areas should be aware of the presence of *B. procyonis* and implement prevention measures accordingly [25,52,60,61]. *Baylisascaris procyonis*, with its potential to cause severe or fatal infections, particularly in children, poses a public health risk [27] that is underestimated in Germany, according to Peter et al. [10]. Nevertheless, cases of Baylisascariasis in humans are rare both in Europe and North America [27,58]. However, serological investigations indicate that subclinical infections may frequently occur in adults in *B. procyonis* areas, suggesting many unreported cases [27,62].

In summary, *B. procyonis* poses a potential One Health threat as it can have a negative health impact on humans and a variety of domestic, zoo, and wildlife species [4,8,27,58,59,61]. In a preventive approach, high raccoon densities should be considered a risk factor; therefore, identifying *B. procyonis* risk areas is highly beneficial [16,27].

#### 4.2.2. *Toxocara canis*, *Capillaria* spp. and *Trichuris* spp.

We detected *T. canis* in the intestine of one raccoon, which is the second description of this nematode in raccoons and the first detection by PCR and Sanger sequencing. Davidson et al. [63] morphologically identified eggs of *Toxocara* spp. and *Toxascaris* spp. in the feces of two Norwegian raccoons. The ascarid eggs collected from the *T. canis*-positive raccoon in the flotation were examined using PCR, revealing the presence of *B. procyonis* and no positive result for *T. canis* eggs. This could be attributed to a non-patent *T. canis* infection or the absence of *T. canis* eggs in the sample used for the flotation and PCR analysis. Consequently, the raccoon’s status as a final host for *T. canis* could not be confirmed with certainty. Nevertheless, the possibility should be considered that raccoons occurring in close proximity to domestic dogs or red foxes (*Vulpes vulpes*) may be involved in disseminating *T. canis*. In dogs, *T. canis* is one of the most common intestinal parasites and is also pathogenic to humans [64]. Fecal examinations in Germany revealed a prevalence of 6.1% in dogs (N = 24,677), with a higher prevalence observed in animals up to six months, being twice as high [65]. In German red foxes, prevalence rates of 43.8% (N = 80) have been reported, making it one of the most commonly detected endoparasites in this species [11]. An infection with *Toxocara* spp. in intermediate hosts follows a nearly equivalent course to *Baylisascaris* spp., although the latter more frequently leads to severe or fatal neural damage [25,27]. Toxocariasis is the most common helminthic zoonosis in industrialized countries, with a human seroprevalence of 6.2% in Europe and 4.8% in Germany [27,64,66]. Peak values of 32.7% were recorded in Ireland and 27.4% in France [66].

We detected eggs of *Capillaria* spp. in one raccoon and *Trichuris* spp. using the ELISA PetChek^TM^ IP assay (IDEXX, Kornwestheim, Germany) in two raccoons’ fecal samples. Both genera have been previously detected in raccoons in Germany, albeit with low prevalence [10,31,47].

No *Trichinella* spp. were found in this study. In Germany, several investigations have been conducted on *Trichinella* spp. in raccoons, but only immunological detection in meat juice has been successful [10,67,68]. This nematode is globally distributed and is regularly found in carnivores and wild boars in Germany [67,68,69]. *Trichinella* spp. have been detected in raccoons in Europe, North America, and Japan [67,68].

### 4.3. Cestodes

In this study, *A*. cf. *incisa/procyonis* was the most commonly detected helminth, with a prevalence of 43.6% among the 101 examined raccoons. The species allocation is uncertain. In total, five species of *Atriotaenia* have been described, four from American procyonids, mustelids, and bats [70]. Relevant here are *A. procyonis* from North American raccoons and *A. incisa* from European badgers. Both species are morphologically indistinguishable and possibly conspecific [43]. In the absence of any gene sequence data prior to our study, we are not in a position to decide whether the taxon we found in our raccoons had been translocated from North America with its host (as with *B. procyonis*), or whether the indigenous badger parasite described as *A. incisa* has expanded its host range to raccoons. Genetic data from North America and Europe will be necessary to draw a conclusion from this. The parasite burden in the affected raccoons is higher compared to all the other parasites found, with 25% of the 44 positive raccoons (n = 11; 95% CI: 13.2–40.3) being heavily infected and 22.7% (n = 10; 95% CI: 11.5–37.8) moderately infected (Figure 3). These high parasite burdens are consistent with previous investigations in Germany [43]. In Europe, *Atriotaenia* spp. has been repeatedly detected in raccoons, with the recent prevalence ranging from 12.0% (N = 175) in Germany to 19.7% (N = 234) in Poland [10,18]. In comparison, the prevalence detected here is more than twice as high. The high prevalence and infestation rates found in this and previous studies in Germany suggest that raccoons are important hosts of this parasite [10]. Significantly higher prevalence rates of up to 86.9% (N = 35) have been reported from raccoons in North America [29,71,72]. There have been relatively few studies on *A*. cf. *incisa/procyonis*, so little is known about its life cycle and pathogenicity [73]. Cyclophyllidean cestodes require intermediate hosts for their development, in the case of *Atriotaenia* sp. most probably coprophagous beetles [72]. Due to raccoons consuming beetles throughout the year, the likelihood of infection is relatively high [20]. Statistically, it was determined that a significantly higher proportion of *A.* cf. *incisa/procyonis*-positive raccoons originated from rural areas. This could be associated with a presumed higher abundance of intermediate hosts in rural areas and the increased utilization of anthropogenic food sources by raccoons in urban areas, which can decrease the diversity of parasites with indirect life cycles [71,74].

We identified *T. martis* for the first time in raccoons. *Taenia martis* is widely distributed in Europe and commonly infects the intestines of various species of Mustelidae [75,76]. In southern Germany, prevalence rates of *T. martis* were found to be 36% (N = 47) in stone martens (*Martes foina)* and 48% (N = 437) in muskrats (*Ondatra zibethicus*) [77]. Whether raccoons could be a new possible final host of *T. martis* with patent infections is unclear since both individuals were immature and only about 3 cm long. However, the fact that the parasite was found in two raccoons from different counties (SHA, OAK) may indicate a broader distribution in the raccoon population of BW. Other taeniids such as *Hydatigera sp.*, *T. crassiceps*, and *T. pisiformis* have already been detected in raccoons [71,78,79]. *Taenia martis* is considered an emerging infectious pathogen because it causes eye and brain infections in humans and can induce severe to fatal infections in nonhuman primates [75,76,79]. It should be noted that out of four described human cases worldwide, three have occurred in Germany [79].

*Mesocestoides* spp. could only be detected at the genus level in one raccoon in this study. The accurate species identification of *Mesocestoides* spp. is difficult due to taxonomic uncertainties within this genus and should, at best, be performed both morphologically and molecularly [80]. In German raccoons, a prevalence of 2.6% (N = 234) to 3.5% (N = 762) has been described so far [10,50]. In comparison, a study from Poland showed that a higher prevalence of 67% (N = 55) can occur [47]. This may indicate that raccoons could likely be final hosts of *Mesocestoides* spp. This genus is distributed worldwide in various carnivorous final hosts and, in individual cases, can also be transmitted to humans [79,80]. Common second intermediate hosts are small mammals, birds, reptiles, and amphibians [47,80], which are also essential sources of feed for raccoons [20].

Comparing the prevalence of the cestode genera found, it should be noted that cestodes using small mammals as intermediate hosts (*T. martis*, *Mesocestoides* spp.) are much less common. Another important zoonotic parasite with such a heteroxenous life cycle, *Echinococcus* spp., was not detected. By now, no *Echinococcus* sp. has been described in raccoons, either in North America or elsewhere. Considering the high prevalence of *Echinococcus multilocularis* in red foxes in our study area, the involvement of raccoons in the life cycle of this parasite appears to be negligible [19,81].

### 4.4. Trematodes

Raccoons have been found to harbor numerous species of trematodes. The use of water-rich environments makes infections with digenetic trematodes likely because raccoons in these habitats may feed on insects, amphibians, mollusks, and fish, all of which are known intermediate hosts of trematodes [20,82]. The low number of trematode infections observed in this study is consistent with the low infection rates in Hessian and Bavarian raccoons, reported by Peter et al. [10]. Several explanations exist, such as the possibility that raccoons serve only as accidental hosts or that trematodes pass through their digestive tract without maturing. Differences in habitat use, such as less use of or access to water-rich areas and thus less contact with potentially infected intermediate hosts, may also play a role. In addition, there are methodological limitations, as described earlier, since flukes quickly decompose through freezing and thawing processes, rendering them undetectable.

Among the examined raccoons, 4% were found to harbor the species *I. hortensis/melis*. In this project, the species *I. melis* and *I. hortensis* were grouped due to their close morphological resemblance, making it impossible to differentiate them without using genetic techniques [83]. Morphologically identifying *Isthmiophora* spp. is challenging because they can vary in morphology within and between species depending on the host [84]. Unlike other representatives in the Echinostomatidae family, *Isthmiophora* spp. exclusively infest mammals’ intestinal tract during their adult phase [85]. So far, over 30 species of vertebrates, including humans, have been identified as final hosts [84,85]. Both *I. melis* and *I. hortensis* have been found in the intestines of raccoons, wild boars (*Sus scrofa*), raccoon dogs (*Nyctereutes procyonoides*), and other wild animals [84,85]. In central Europe, the prevalence of *Isthmiophora* spp. infections in raccoons has been reported to range from 4.8% (N = 62; Czechia) to 50.3% (N = 175; Germany and Poland) [18].

In 1% of the examined raccoons, another potentially human pathogenic trematode species, *Plagiorchis muris*, commonly found in freshwater ecosystems across Europe, was identified based on its morphology [86]. *Plagiorchis muris* has previously been detected in raccoons, including in Germany, with a prevalence of 0.9% (N = 234) [10,78,86]. This parasite is typically found in the small intestine of rodents, birds, reptiles, insectivores, and omnivores [87]. Although human infections have been described, the risk for humans is minimal due to the trophic transmission primarily through raw fish [87].

Morphologically, *Brachylaima* spp. were also identified in almost 2% of the raccoons. This genus has been frequently described in raccoons, with a prevalence of 3.8% (N = 234) in central Germany [10,82].

## 5. Conclusions

In summary, according to our findings, the raccoons’ propensity for spreading and synanthropic habits may lead to environmental pollution in BW with pathogens, including helminths [9]. Consequently, raccoons may serve as a transmission link between various animal groups, including wildlife, domestic and zoo animals, and humans, thereby elevating the risk of helminthic infections [3,6,12,14]. The detection of the parasite variety and abundance of potentially zoonotic helminths, including *B. procyonis*, *T. canis*, *T. martis*, *Mesocestoides* spp., *Plagiorchis muris,* and *I. hortensis/melis* are relevant considering One Health aspects. The relatively low infestation levels with individual helminths and the co-infections of various helminth groups, compared to the main distribution areas of raccoons in Germany, provide evidence for the raccoon’s early stage of spread in BW [10,49]. Despite this, the present study shows a relatively high diversity of helminth fauna in raccoons from BW, indicating an existing interaction in the life cycles of these helminths in BW. Anthropogenic alterations, along with host- and pathogen-specific factors, influence the dynamics of environmental pathogen pollution and helminthic transmissions among wildlife species, domestic animals, and humans [3,6,9]. Urbanization and the climate crisis, in particular, can have a significant impact on the epidemiology of helminths, such as *B. procyonis*, and the occurrence and spread of intermediate or final hosts, such as raccoons [2,4,5,19,22,88]. As raccoons continue to expand their population in BW, they are likely to integrate into new distribution areas and serve as new hosts for native parasites and pathogens [9,10,12,15,18].

Therefore, in regions with high biodiversity, endangered species, and human populations, it could be advisable to implement measures to deter, control, or eradicate raccoons to minimize their potential negative impact as IAS and their contribution to pathogen pollution [9,21,24]. However, in this regard, it should be considered that increasing mortality through hunting will raise the turnover rates of the population and disease transmission due to high offspring rates or migrating raccoons, which may be counterproductive [20]. Additionally, in alignment with the One Health approach, the four fundamental elements of prediction, prevention, diagnosis, and intervention are imperative [4]. Prevention measures (e.g., hygiene concepts, public education, anthelminthic management in pets, baiting programs, and raccoon management) are essential to mitigate the negative impact of helminth infections in raccoons on both human and animal health [2,8,25,82]. The regular removal of latrines and heat-based sterilization methods of the surroundings should be employed in raccoon-inhabited areas to minimize environmental contamination with helminth eggs [8,25,27].

Our results highlight the importance of wildlife disease surveillance systems as part of a One Health approach [2,3,5,6,9,54]. Exploring risk factors in wildlife final and intermediate hosts and the environment will provide a better understanding of the epidemiology of helminths, the role of wildlife, and the potential negative consequences of infectious pathogens [4,54]. This exploratory study provides the framework for selective prevalence studies with larger sample sizes, latrine monitoring, or research on intermediate hosts in the study area to obtain a more profound insight into the infection situation, environmental contamination, e.g., with *B. procyonis*, and infection risk for humans and animals with helminths. Moreover, similar studies in other parts of Germany and Europe would improve our understanding of the differences in the occurrence of helminths in raccoons.

## Figures and Tables

**Table 1:** PCR primers used to detect Cestodes and Nematodes.

Target Gene		Primer	Primer-Sequence	PCR Product Length
**Cestodes** ^1^				
*nad1*	first PCR	*nad1* Forward	5′-TGATGATTTGTCTAGTC-3′	∼990 bp
		*nad1* Reverse	5′-TTCTTGAAGTTAACAGC-3′	
	nested PCR	*nad1* Fornest	5′-GATTTGTCTAGTCATAGATG-3′	∼980 bp
		*nad1* Revnest	5′-CTTGAAGTTAACAGCATCACG-3′	
*cox1*	first PCR	*cox1H* Forward	5′-WATAAAGGTTTRTTATTTGCTATG-3′	∼930 bp
		*cox1H* Reverse	5′-ATCHAWTAAGCATGATGCAAAAGG-3′	
	nested PCR	*cox1H* Fornest	5′-TATGTTTTCAATAGTBTGTTTAGG-3′	∼890 bp
		*cox1H* Revnest	5′-CATGATGCAAAAGGCAAAWAAACC-3′	
**Nematodes** ^2^				
*nad1*	first PCR	Toxo Forward	5′-ATTGCTTTTATTACTTTGTATGAGC-3′	∼480 bp
		Toxo Reverse	5′-GCAAATAAATTACCAACAAACTC-3′	
	nested PCR	Toxo Fornest	5′-CCTAATAAGGTTAGTTTTAT-3′	∼380 bp
		Toxo Revnest	5′-AAAAACAAAATATGTTAACATG-3′	

^1^ Dumendiak, S., unpublished; ^2^ Wassermann, M., unpublished. Fornest, forward primers for nested PCR; Revnest, reverse primers for nested PCR.

**Table 2:** Results. The table shows the absolute and relative results with 95% confidence intervals according to the individual categories. The relative frequency describes the proportion of positive results within each category.

	% (n, CI 95%)							
Parameters	Raccoons (n)	Helminth Positives	Nematodes	*B. procyonis*	Cestodes	*A.* cf. *incisa/procyonis*	Trematodes	*I. hortensis/melis*
**Total**	101	72.3 (73; 62.5–80.7)	31.7 (32; 22.8–41.7)	28.7 (29; 20.1–38.6)	46.5 (47; 36.5–56.7)	43.6 (44; 33.7–53.8)	15.8 (16; 9.3–24.4)	4 (4; 1.1–9.8)
**Years**								
2019	32.7 (33; 23.7–42.7)	69.7 (23; 51.3–84.4)	24.2 (8; 11.1–42.3)	21.2 (7; 9.0–38.9)	48.5 (16; 30.8–66.5)	45.5 (15; 28.1–63.6)	18.2 (6; 7.0–35.5)	6.1 (2; 0.7–20.2)
2020	67.3 (68; 57.3–76.3)	73.5 (50; 61.4–83.5)	35.3 (24; 23.7–47.2)	32.4 (22; 21.5–44.8)	45.6 (31; 33.5–58.1)	42.6 (29; 30.7–55.2)	14.7 (10; 7.3–25.4)	2.9 (2; 0.4–10.2)
**Area**								
rural	32.7 (33; 23.7–42.7)	75.8 (25; 55.6–87.1)	27.3 (9; 13.3–45.5)	24.2 (8; 11.1–42.3)	63.6 (21; 45.1–79.6) *	57.6 (19; 39.2–74.5) *	12.1 (4; 3.4–28.2)	3 (1; 0.1–15.8)
urban	67.3 (68; 57.3–76.3)	70.6 (48; 58.3–81.0)	33.8 (23; 22.8–46.3)	30.9 (21; 20.2–43.3)	38.2 (26; 26.7–50.8) *	36.8 (25; 25.4–49.3) *	17.6 (12; 9.5–28.8)	4.4 (3; 0.9–12.4)
**Sex**								
♀	38.6 (39; 29.1–48.8)	69.2 (27; 52.4–83.0)	25.6 (10; 13.0–42.1)	23.1 (9; 11.1–39.9)	51.3 (20; 34.8–67.6)	51.3 (20; 34.8–67.6)	15.4 (6; 5.9–30.5)	0 (0; 0–9.0)
♂	61.4 (62; 51.2–70.9)	74.2 (46; 61.5–84.5)	35.5 (22; 23.7–48.7)	32.3 (20; 20.9–45.3)	43.5 (27; 31.0–56.7)	38.7 (24; 26.6–51.9)	16.1 (10; 8.0–27.7)	6.5 (4; 1.8–15.7)
**Age**								
>1 a	54.5 (55; 44.2–64.4)	80.0 (44; 67.0–89.6)	34.5 (19; 22.2–48.6)	32.7 (18; 20.7–46.7)	58.2 (32; 44.1–71.3)	52.7 (29; 38.8–66.3)	14.5 (8; 6.5–26.7)	3.6 (2; 0.4–12.5)
<1 a	45.5 (46; 35.6–55.8)	63.0 (29; 47.5–76.8)	28.3 (13; 16.0–43.5)	23.9 (11; 12.6–38.8)	32.6 (15; 19.5–48.0)	32.6 (15; 19.5–48.0)	17.4 (8; 7.8–31.4)	4.4 (2; 0.5–14.8)
**Age–Sex Class**								
>1 a + ♀	15.8 (16; 9.3–24.4)	81.2 (13; 54.4–96.0)	31.2 (5; 11.0–58.7)	31.2 (5; 11.0–58.7)	68.8 (11; 41.3–89.0)	68.8 (11; 41.3–89.0)	12.5 (2; 1.6–38.3)	0 (0; 0–20.6)
>1 a + ♂	38.6 (39; 29.1–48.8)	79.5 (31; 61.5–89.2)	35.9 (14; 21.2–52.8)	33.3 (13; 19.1–50.2)	53.8 (21; 37.2–69.9)	46.2 (18; 30.1–62.8)	15.4 (6; 5.9–30.5)	5.1 (2; 0.6–17.3)
<1 a + ♀	22.8 (23; 15.0–32.2)	60.9 (14; 38.5–80.3)	21.7 (5; 7.5–43.7)	17.4 (4; 5.0–38.8)	39.1 (9; 19.7–61.5)	39.1 (9; 19.7–61.5)	17.4 (4; 5.0–38.8)	0 (0; 0–14.8)
<1 a + ♂	22.8 (23; 15.0–32.2)	65.2 (15; 41.7–83.6)	34.8 (8; 16.4–57.3)	30.4 (7; 13.2–52.9)	26.1 (6; 10.2–48.8)	26.1 (6; 10.2–48.8)	17.4 (4; 5.0–38.8)	8.7 (2; 1.1–28.0)

* significant relationships calculated with Fisher’s exact test with a significance level of *p* ≤ 0.025. a, year. *B. procyonis*, *Baylisascaris procyonis*; *A.* cf. *incisa/procyonis*, *Atriotaenia* cf. *incisa/procyonis*; *I. hortensis/melis*, *Isthmiophora hortensis/melis;* ♀, female; ♂, male.

**Table 3:** Co-infections of nematodes, cestodes, and trematodes. This table shows the absolute and relative co-infection results with 95% confidence intervals for the individual categories. The relative frequency describes the proportion of co-infections within the positive raccoons of each category.

		% (n Positives, CI 95%)				
Parameters	n Positives	Coinfections	Nem/Cest	Nem/Trem	Cest/Trem	Nem/Cest/Trem
**Total**	73	32.9 (24; 22.3–44.9)	21.9 (16; 13.1–33.1)	2.7 (2; 0.3–9.5)	2.7 (2; 0.3–9.5)	1.4 (1; 0–7.4)
**Years**						
2019	23	34.8 (8; 16.4–57.3)	17.4 (4; 5.0–38.8)	0 (0; 0–14.8)	4.3 (1; 0.1–21.9)	4.3 (1; 0.1–21.9)
2020	50	32 (16; 19.5–46.7)	24 (12; 13.1–38.2)	4 (2; 0.5–13.7)	2 (1; 0.1–10.6)	0 (0; 0–7.1)
**Area**						
rural	25	40 (10; 21.1–61.3)	28 (7; 12.1–49.4)	0 (0; 0–13.7)	5.9 (2; 0.7–19.7)	0 (0; 0–13.7)
urban	48	29.2 (14; 17.0–44.1)	18.8 (9; 9.0–32.6)	4.2 (2; 0.5–14.3)	0 (0; 0–5.3)	2.1 (1; 0.1–11.1)
**Sex**						
♀	27	33.3 (9; 16.5–54.0)	25.9 (7; 11.1–46.3)	3.7 (1; 0.1–19.0)	3.7 (1; 0.1–19.0)	0 (0; 0–12.8)
♂	46	32.6 (15; 19.5–48.0)	19.6 (9; 9.4–33.9)	2.2 (1; 0.1–11.5)	2.2 (1; 0.1–11.5)	2.2 (1; 0.1–11.5)
**Age**						
>1 a	44	36.4 (16; 22.4–52.2)	27.3 (12; 15.0–42.8)	4.5 (2; 0.6–15.5)	2.3 (1; 0.1–12.0)	0 (0; 0–8.0)
<1 a	29	27.6 (8; 12.7–47.2)	13.8 (4; 3.9–31.7)	0 (0; 0–11.9)	3.4 (1; 0.1–17.8)	3.4 (1; 0.1–17.8)
**Age–Sex Class**						
>1 a + ♀	13	38.5 (5; 13.9–68.4)	30.8 (4; 9.1–61.4)	7.7 (1;0.2–36.0)	0 (0; 0–24.7)	0 (0; 0–24.7)
>1 a + ♂	31	35.5 (11; 19.2–54.6)	25.8 (8; 11.9–44.6)	3.2 (1; 0.1–16.7)	3.2 (1; 0.1–16.7)	0 (0; 0–11.2)
<1 a + ♀	14	28.6 (4; 8.4–58.1)	21.4 (3; 4.7–50.8)	0 (0; 0–23.2)	7.1 (1; 0.2–33.9)	0 (0; 0–23.2)
<1 a + ♂	15	26.7 (4; 7.8–55.1)	6.7 (1; 0.2–31.9)	0 (0; 0–21.8)	0 (0; 0–21.8)	6.7 (1; 0.2–31.9)

Nem, nematodes; Cest, cestodes; Trem, trematodes; a, year; ♀, female; ♂, male.

## Data Availability

The datasets generated during and/or analyzed during the current study are available from the corresponding author on reasonable request.
